# Intimate partner violence, suicide and self-harm in Sri Lanka: Analysis of national data

**DOI:** 10.1371/journal.pone.0298413

**Published:** 2024-03-21

**Authors:** Piumee Bandara, Andrew Page, Thilini Rajapakse, Duleeka Knipe

**Affiliations:** 1 Population Health Sciences, Bristol Medical School, University of Bristol, Bristol, United Kingdom; 2 South Asian Clinical Toxicology Research Collaboration, Faculty of Medicine, University of Peradeniya, Peradeniya, Sri Lanka; 3 Translational Health Research Institute, Western Sydney University, New South Wales, Australia; 4 Department of Psychiatry, Faculty of Medicine, University of Peradeniya, Peradeniya, Sri Lanka; University of Ghana College of Humanities, GHANA

## Abstract

There is increasing evidence from South Asia and internationally that intimate partner violence (IPV) is strongly associated with self-harm, however its association with suicide and self-harm has not been extensively examined, nor has this relationship been explored at a national level. Using national datasets, area-level variation in IPV, suicide and self-harm in Sri Lanka were examined. In addition, the association between individual level exposure to past-year IPV and non-fatal self-harm by any household member were explored in a series of multi-level logistic regression models, adjusting for age. Similar patterns in the distribution of suicide and IPV were found, with higher rates evident in post-conflict districts, specifically Batticaloa, Kilinochchi, and Mullaitivu. Experience of past year IPV and its various forms were strongly associated with household-level self-harm in the past year (adjusted odds ratio [AOR] = 3.83 95% CI 2.27–6.46). A similar magnitude was found for physical/sexual abuse (AOR 5.17 95% CI 2.95–9.05) and psychological abuse (AOR 4.64 95% CI 2.50–7.00). A dose-response association was also evident for frequency of abuse, with an increasing risk of household-level self-harm for women reporting abuse ‘less often’ (AOR 2.95 95% CI 1.46–5.92), and abuse experienced ‘daily, weekly, or monthly’ (AOR 4.83 95% CI 2.59–9.00), compared to no abuse. This study contributes to a growing body of evidence on the relationship between IPV and suicidal behaviour in South Asia. Addressing IPV and its various forms should be a priority for suicide prevention in Sri Lanka, alongside trauma-informed approaches in post-conflict settings.

## Introduction

Intimate partner violence (IPV) and suicidal behaviour are significant contributors to mortality and morbidity worldwide. Over 700,000 people die by suicide, every year [1]. The majority of these deaths occur in low- and middle-income countries (LMIC), accounting for 77% of total deaths, and 29% within South Asia alone [1]. For every suicide, there is an estimated 10–20 self-harm acts, [2] equating to approximately four million acts of self-harm every year in South Asia.

Despite carrying a considerable share of the global suicide and self-harm burden, research is largely based in high-income countries [3]. Understanding the epidemiology and aetiology of suicide and self-harm in LMIC is needed to appropriately inform suicide prevention strategies. A 2019 systematic review and meta-analysis showed that compared to high-income countries, psychiatric morbidity appeared to be lower among those who engaged in self-harm in LMIC [4]. This suggests that a broader approach to suicide prevention is needed that addresses other social risk factors, including poverty and interpersonal conflict [4, 5].

IPV is particularly acute in South Asia where 35% of ever-partnered women reported experiencing IPV in their lifetime, compared to 20% in Western Europe and 21% in high-income Asia Pacific [6]. Within Sri Lanka, an estimated 40% (95% CI 38% - 42%) of women aged 15 years or older reported experiencing physical, sexual, emotional, and/or economic violence and/or controlling behaviours by a partner in their lifetime [7]. As an emerging economy, Sri Lanka is undergoing a period of increasing urbanisation and transition in social and gender norms. Furthermore, Sri Lanka is recovering from the effects of a protracted civil war which ended in 2009 and continues to report high rates of suicide and self-harm, particularly among young females [8–11]. Populations exposed to conflict are known to experience higher rates of unemployment, poverty, and social dysfunction [12–14]. Co-occurring issues of poverty, a breakdown in social norms, and the normalisation of violence in post-conflict settings creates an environment where harmful behaviours such as alcohol misuse, childhood abuse, and IPV are more likely to occur, as has been reported in community surveys from Sri Lanka and Afghanistan [15].

Conflict between an intimate partner and family members has been identified as a key precipitating trigger for suicidal behaviour across Asia, particularly among females [9, 16–18]. IPV has been shown to be strongly associated with suicidal behaviour, although much of the evidence base is concentrated in HIC [19–21]. A WHO study of violence against women in 15 countries (eight of which were LMIC) found women who had experienced physical or sexual violence, or both in their lifetime, were almost four times more likely to attempt suicide, when compared to women who had never experienced partner violence [22]. Studies from South Asia also consistently report high prevalence of domestic violence among hospital presenting self-harm cases, with estimates as high as 72% in Nepal [23], 73% in Afghanistan [24], and between 46% [25] to 70% [26] in India.

In Sri Lanka, the relationship between IPV and suicidal behaviour has been studied to a much lesser extent. There is evidence of strong associations between domestic violence and self-harm, although these studies were limited to the hospital and did not examine suicide [27, 28]. Given Sri Lanka’s unique history with civil war and its associated economic, social, and health implications, further understanding of the relationship between IPV with suicide and self-harm is needed at the national level. Furthermore, given the high levels of IPV in South Asia, and high rates of self-harm and suicide, particularly among young females in South Asia, further understanding of this relationship is needed.

To our knowledge, this is the first study to examine the relationship between IPV, suicide, and self-harm using national data. In 2016, the Sri Lanka Demographic and Health Survey (SLDHS) introduced, for the first time, questions on experience of IPV in the previous 12-months among ever-partnered females aged 15–49 years. This is the first nationally representative population-based survey of IPV conducted in Sri Lanka. Using the SLDHS dataset, together with suicide data sourced from the Sri Lankan Department of Police, we aimed to:

1. Explore regional patterns in the distribution of IPV, suicide, and self-harm in Sri Lanka.
2. Examine associations between exposure to past-year IPV (and its various forms and frequency of abuse) and occurrence of past-year self-harm within the household.
3. Examine between exposure to past-year IPV (and its various forms and frequency of abuse) and occurrence of past-year self-harm/suicide (combined) within the household.

## Methods

### Study setting

Sri Lanka is a lower-middle income island nation situated off the South-East coast of India, with a population of 20.4 million people [29]. The majority of Sri Lankans identify as Sinhalese (75%), followed by Tamil (11%) and Moor (9%). The predominant religion practiced in Sri Lanka is Buddhism (70.1%), with most Sinhalese people identifying as Buddhist, most Tamils as Hindu, and Moors as Muslim. The majority of the population (77%) reside in rural areas, followed by urban areas (18%), and the estate sector (4%) [30]. The estate sector is defined by its agricultural plantations (mostly tea, rubber and coconut) and is largely populated by Indian Tamil plantation workers and their families who arrived in Sri Lanka as indentured labourers from South India under British Colonial rule in the 19^th^ century [14, 29]. Between 1983–2009, Sri Lankan government forces and Tamil separatists engaged in a violent civil war with the conflict predominantly in the Northern (94% Tamil population) and Eastern (40% Tamil and 37% Moor population) provinces [29]. The latter was additionally the province worst affected by the 2004 tsunami in terms of human lives lost and households damaged [31].

### Data

Secondary analysis were conducted on publicly available anonymised data from two sources, the Sri Lanka 2016 Demographic and Health Survey and Sri Lanka Department of Police. An overview of the data sources are provided in S1 Fig and detailed further below.

#### Sri Lanka 2016 Demographic and Health Survey

Between May to November 2016, the Sri Lankan Department of Census and Statistics (DCS) undertook the national SLDHS. Detailed sampling methods are described in the 2016 SLDHS Report [32]. In brief, the survey used a two-stage stratified cluster sampling technique. At the first stage, 2500 census blocks (clusters) were stratified by district and then by sector (urban, rural and estate). In stage two, 12 households from each cluster (10 households in the Western province) were selected by equal probability systematic sampling. A total of 18,302 ever-partnered women aged 15–49 years were interviewed in private.

*Intimate partner violence*. Data on IPV were obtained from the IPV module that was introduced for the first time in the 2016 SLDHS. In accordance with the WHO guidelines for the ethical collection of information on IPV, only one eligible ever-partnered woman (i.e., married, living with a partner, or separated, divorced, or widowed) per household was randomly selected for the IPV module resulting in a sample of 16,390 females. Questions related to frequency of experience of physical, sexual, emotional and psychological IPV in the previous 12-months preceding the survey. IPV exposure was defined as experiencing any IPV in the past-year versus no past-year exposure. Given different forms of abuse and the frequency of IPV present varying levels of risk [19], additional IPV variables were generated to compare the differential effects by abuse type and frequency of IPV on self-harm. Physical violence was defined as women reporting being ‘slapped’, ‘pushed’, ‘beaten with an object’, ‘strangled’ or ‘burned’ in the past year. Sexual violence was defined as ‘forced sex’. Due to the high proportion of sexual violence cases that occur with physical violence (74%), a composite physical/sexual violence variable was created. Physical/sexual violence was categorised as any act of physical and/or sexual violence with or without psychological violence in the past year versus no past-year IPV. Psychological violence was categorised as women who reported being ‘prevented from leaving home’, or experiencing ‘belittlement or serious offence’, with or without physical and/or sexual violence in the past year.

*Non-fatal self-harm*. Self-harm and suicide occurring within the past year were collected from females via the question: “Did any member of your household try to commit suicide in the last 12 months?” followed by the question “Did the person die?” The identity of the household member who engaged in suicidal behaviour was not ascertained from female respondents. Given self-harm is a distinct behaviour to suicide, for definitional clarity, the ten respondents (0.06% of the sample) who reported that the suicide attempt by a household member ended in death were excluded from the main analysis. A sensitivity analysis was conducted using a combined suicide and self-harm variable, and differences to the main analysis were compared.

#### Department of Police, Division of Statistics, Sri Lanka–suicide count data

Publicly available data on suicide counts for the year 2018 were obtained from the Department of Police, Division of Statistics, Sri Lanka. The number of suicides were reported according to 43 police divisions across the country. Multiple police divisions are situated within a given government administrative district, however, no police division crossed district boundaries. The total sum of suicides were aggregated to the administrative district level similar to a previous study using Sri Lankan police suicide data [33]. Data were not available on age. To enhance statistical power, male and female suicide counts were combined.

### Statistical analysis

#### Regional estimates of IPV, self-harm and suicide

Geographical variation in the prevalence of IPV and crude rate of suicide were visualised through choropleth maps. District-level prevalence of IPV was calculated using the SLDHS weighted count of IPV as the numerator and weighted count of total respondents for each district as the denominator. IPV prevalence were defined into five equal intervals as previously reported [34]. Crude rate of self-harm by a household member were calculated as the weighted number of SLDHS respondents who reported self-harm by a household member in the previous 12-months, divided by the total weighted number of survey respondents of a given province. Given the numbers at district-level were too low to estimate meaningful rates with confidence intervals, we deviated from our analysis plan [35], and presented crude self-harm rate by province rather than district. Crude suicide rates for each district were calculated by dividing the total number of suicides for the districts, by the 2017 mid-year Census district estimated population, and rates presented using the equal interval method.

#### Multilevel logistic regression models

The association between IPV and household-level self-harm were explored through logistic regression models using complete DHS data for IPV, age, and self-harm variables, according to our pre-registered analysis plan [35]. The final analytical sample comprised 16,390 females aged 15–49 years. Data on IPV were collected from females, nested within clusters. Participants nested within the same cluster are likely to share similar characteristics than participants in a different cluster. To account for the hierarchical structure of the data and potential clustering effects on the outcome of interest, multilevel (two-level) logistic regression models were fit with households at level one and clusters at level two. Compared to single level regression modelling, this modelling enables the estimation of how much of the total variance in the outcome is attributed to higher level (cluster) units [36]. First, an empty (no covariates) random-intercept model was generated and the proportion of unadjusted total variance in IPV attributable to cluster-level variance (variance partition coefficient [VPC]) was estimated (VPC = 20%) using the latent variable method described by Merlo et al., 2006 [36]. The main model minimally adjusted for age as a confounder, previously identified as a correlate of IPV and self-harm in this setting [37, 38]. Associations for different types of abuse (physical/sexual abuse and psychological abuse) and dose-response effects (i.e., frequency of abuse) were also examined. Given the low count of self-harm (n = 82), a sensitivity analysis using a combined self-harm and suicide outcome variable (n = 92) was conducted and differences in the associations compared to the main analysis. All analyses were conducted in Stata (version 16.1, Stata Corp, College Station, TX, USA). The choropleth maps were generated using the ‘spmap’ command. The ‘melogit’ command was used for multilevel regression analyses.

## Results

### Missing data

Complete data was provided on suicide deaths by the Department of Police, Division of Statistics, Sri Lanka. Ten percent of women eligible for the IPV module of the SLDHS did not respond/were excluded on the basis of privacy issues (7%), refusal to participate (2%), and incompletion of IPV module (<1%) [32]. Ten respondents (0.06%) who reported a suicide death by a household member were excluded from the main analysis. No missing SLDHS data were identified for age. Younger participants (15–34 years) had slightly higher levels of missing data (12%) compared to older participants aged 35–49 years (9%). There was no considerable variation in the level of missing data for self-harm and IPV variables.

### Regional variation of suicidal behaviour and IPV

**Fig 1:**
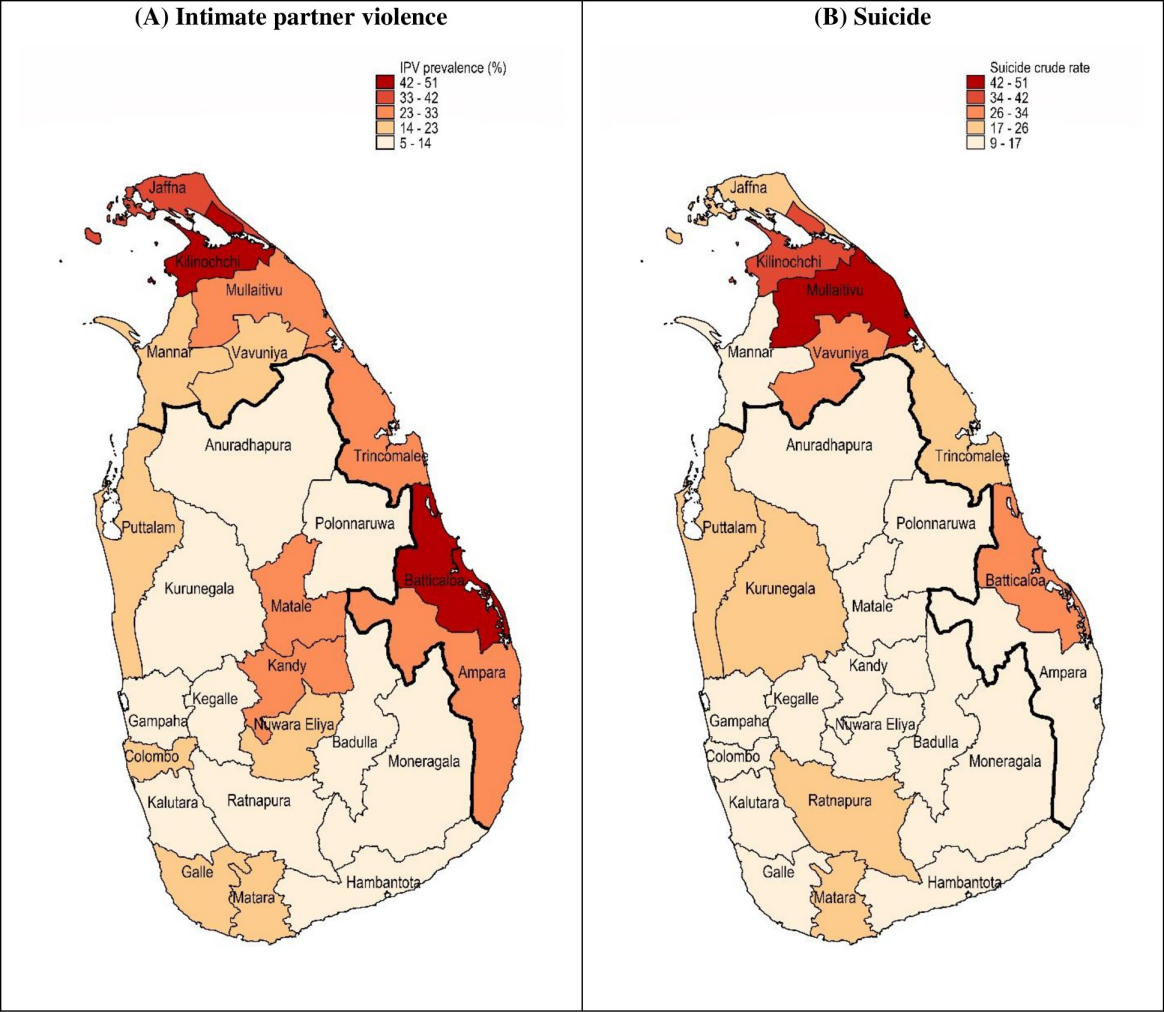
Geographical variation of intimate partner violence (IPV) prevalence and crude suicide rate. (A) Past-year IPV prevalence (Data: 2016 Sri Lanka Demographic and Health Survey data); and (B) Crude suicide per 100,000 population (Data: 2018 Department of Police, Division of Statistics, Sri Lanka Police). Districts above the bold line are post-conflict areas.

**Fig 2:**
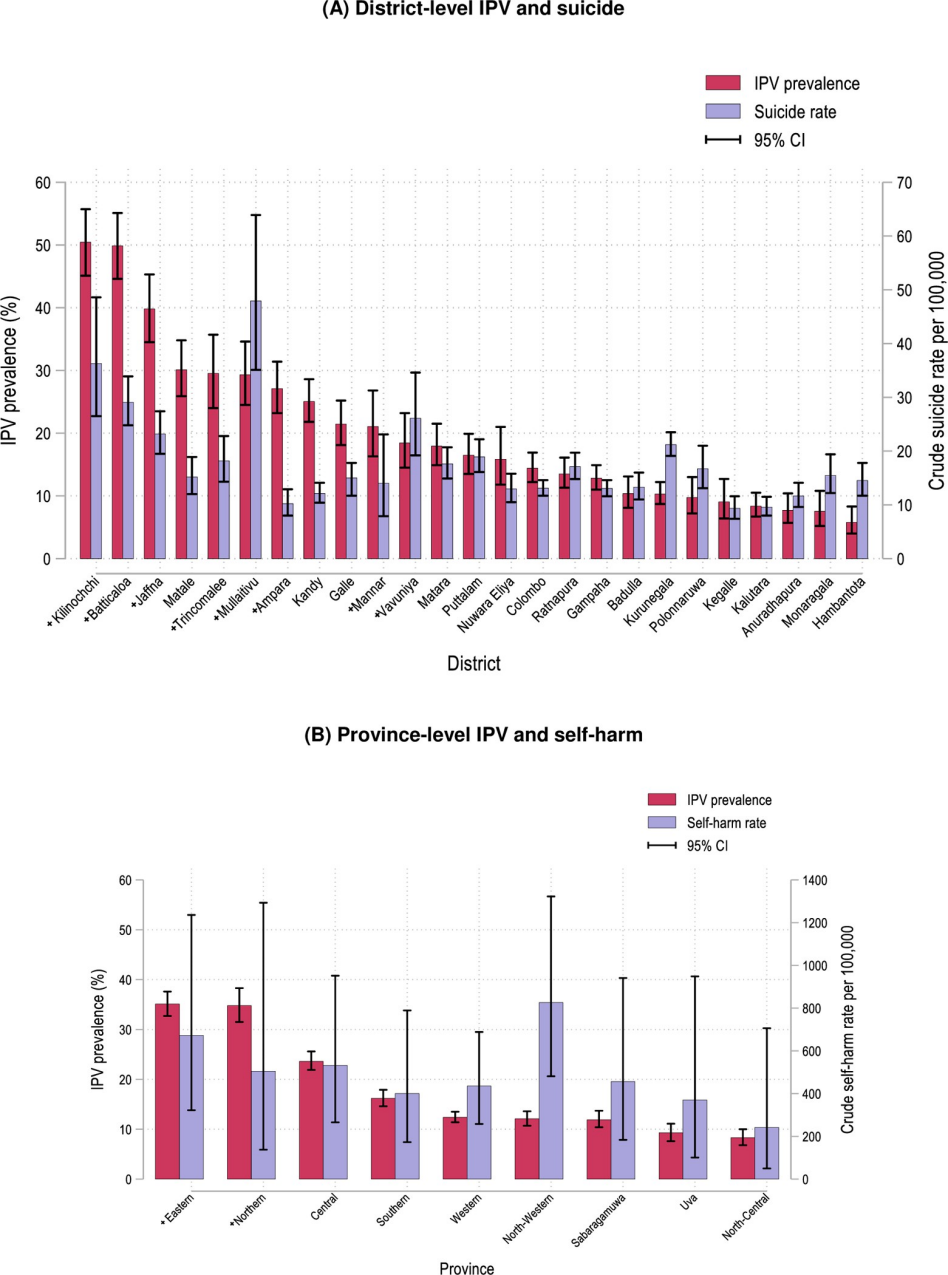
Geographical variation in intimate partner violence (IPV), suicide, and self-harm. (A) District-level past-year IPV prevalence (Data: 2016 Sri Lanka Demographic and Health Survey) and crude suicide per 100,000 population (Data: 2018 Department of Police, Division of Statistics, Sri Lanka Police); (B) Province-level past-year IPV prevalence and crude self-harm rate per 100,00 (Data: 2016 Sri Lanka Demographic and Health Survey). (+) Symbol represents post-conflict areas.

Crude rate of suicide and prevalence of IPV were highest in the Northern and Eastern post-conflict districts (Figs 1 and 2A). For suicide, the highest crude rates were found in the Northern districts of Mullaitivu (48/100,000 population 95% CI 35-64/100,000) and Kilinochchi (36/100,000 95% CI 27-49/100,000), and the Eastern Batticaloa district (29/100,000 95% CI 25-34/100,000) (Figs 1 and 2A and S1 Table). Similarly, for IPV, the highest prevalence was found in the Northern district of Kilinochchi (50% 95% CI 45% - 56%) and Eastern district of Batticaloa (50% 95% CI 45% - 55%) (Fig 2A and S1 Table). High rates of IPV were also found in Matale (30.1% 95% CI 25.9–34.8) and Kandy (25% 95% CI 22% - 29%), districts situated in the Central province. There was no considerable variation in the rates of self-harm by province (Fig 2B), although caution should be exercised given low counts.

### Individual-level IPV exposure and self-harm by a household member

Strong associations, adjusting for age, were found for individual-level exposure to IPV and household-level self-harm, with women experiencing IPV in the past year 3.83 times (95% CI 2.27–6.46) more at risk of reporting self-harm in the household in the previous 12 months, compared to women with no past year history of IPV (Table 1). Similar strong associations were found for physical/sexual abuse (OR 5.17 95% CI 2.92–8.46) and psychological abuse (OR 4.64 95% CI 2.50–7.00). A dose-response association was also evident for frequency of abuse, with an increasing risk of reporting self-harm in the household for women experiencing abuse ‘less often’ (OR 2.95 95% CI 1.46–5.92), and ‘daily, weekly, or monthly’ in the past year (OR 4.83 95% CI 2.59–9.00) (Table 1). Results from the sensitivity analysis using the combined self-harm and suicide variable were consistent with the main analysis (S2 Table).

**Table 1:** Self-harm by a household member in Sri Lanka: Weighted distribution and adjusted associations with ever-partnered women aged 15–49 years reporting intimate partner violence (IPV) in the past year.

	Past year self-harm by household member		
Past-year IPV	Yes	No	Adjusted odds ratio^a^ (95% CI)
	N (%)	N (%)	
**Any abuse**			
No	38 (56.6)	13 587 (83.3)	1.00
Yes	29 (43.4)	2730 (16.7)	3.83 (2.27–6.46)
**Physical/sexual abuse**			
No	38 (62.3)	13 587 (89.5)	1.00
Yes	23 (37.7)	1590 (10.5)	5.17 (2.95–9.05)
**Psychological abuse**			
No	38 (57.8)	13 587 (86.3)	1.00
Yes	28 (42.2)	2163 (13.7)	4.64 (2.50–7.00)
**Frequency of abuse**			
None	38 (56.6)	13 587 (83.3)	1.00
Less often	12 (17.8)	1462 (9.0)	2.95 (1.46–5.92)
Daily, weekly, or monthly	17 (25.6)	1268 (7.8)	4.83 (2.59–9.00)

^a^Adjusting for age. CI = Confidence interval.

## Discussion

Findings from the current study highlight similar patterns in the distribution of suicide and IPV. Higher rates were evident in the post-conflict districts of Killinochchi, Mullaitivu, and Batticaloa. The high rates of IPV and suicidal behaviour found in post-conflict areas in the present study is supported by reports conducted in the region and studies conducted in post-war and post-natural disaster settings [15, 39–43]. Although limited to the area-level, evidence of an association between IPV and suicide is supported by a psychological autopsy study of suicides conducted in three rural and semi-rural districts of Sri Lanka which showed almost half (48.6%) of all suicides reviewed had experienced abuse by a family member [44]. Furthermore, a systematic review of interpersonal violence and female suicide showed up to 51.2% of cases had experienced interpersonal violence [45].

The higher rates of IPV found in post-conflict regions may be explained by the prolonged exposure to collective violence which in turn leads to the normalisation of violence as an acceptable method of conflict resolution [39, 46]. In addition, mental health trauma associated with exposure to conflict can manifest as aggressive behaviours [12]. The protracted civil war in Sri Lanka together with the 2004 tsunami has had considerable social and economic consequences on the population, particularly for those living in the Northern and Eastern provinces. Batticaloa (located in the Eastern province) has relatively poor educational outcomes and household wealth [34]. These socio-economic conditions may explain the higher rates of IPV and suicide in this district as economic insecurity is likely to contribute to interpersonal conflict [34, 46, 47], psychological distress and self-harm [48, 49]. Furthermore, death and disability among males has shifted the focus on females to be more economically active and in some cases the primary economic providers [39]. This represents a destabilisation in gender norms, in which males may feel the need to reassert power and control through abuse [46, 50].

High rates of IPV and suicide were also found in the post-conflict districts of Killinochchi and Mullaitivu. In the final year of the civil war (2009), conflict was concentrated in these districts [51]. The greater exposure to violence in these areas may explain the higher rates of both IPV and suicide, compared to other post-conflict areas. It is possible females living in these communities may also be more accustomed to being asked questions around IPV given the history of research and international and local NGO presence in the area during and following the end of the civil war. As such, respondents may be more forthcoming in disclosing abuse.

At the household level, experience of any past year IPV and its various forms were strongly associated with self-harm by a household member(OR 3.9), and this relationship was dose-dependent, with a higher frequency of abuse increasing likelihood of reporting self-harm. This is consistent with studies from LMIC which show a similar strength in the association between exposure to violence and self-harm, with odds ratio estimates ranging between 3.8 to 7.2 [22, 24, 27, 52, 53]. In Kandy, Sri Lanka, a similar order of magnitude to the current study was found, with women four times more likely to self-poison (95% CI 1.6, 4.8) if exposed to domestic violence in the past year [27]. The association between physical/sexual violence and self-harm is also supported by studies in LMIC settings [52, 54, 55]. In the current study, the magnitude of the association for physical/sexual abuse and psychological abuse did not differ considerably. There is evidence that physical violence is concomitant with emotional, psychological, and sexual abuse, cumulatively increasing the risk of suicide attempts [19, 56]. Much of the research on IPV in LMICs has centred on physical violence against women, our study highlights the need to prioritise other forms of abuse such as psychological abuse in research and prevention efforts. There is also a need to advance research on violence against men and its relationship with suicidal behaviour.

In the context of co-occurring issues of IPV, shifting gender roles, economic stress [34], alcohol misuse (which may be more normalised in areas of poverty [57, 58]), and war/natural disaster-related trauma, it is possible suicidal behaviour may be a response and reaction to compounding stressors [17, 48, 59, 60]. While IPV and suicidal behaviour is more likely to occur in the context of socioeconomic disadvantage [34, 49] and post-conflict environments, the complex interplay of poverty, exposure to violence, and more proximal household environment (e.g., alcohol misuse) and psycho-social factors requires further investigation. Prospective and qualitative studies to further understand the complex mechanisms associating IPV, suicide, and self-harm are needed.

Broader multi-level and multi-sectoral approach to suicide and IPV prevention is needed in Sri Lanka given the multiple intersecting and compounding risk factors operating at the individual, household, community, and societal level. Community-based programs addressing harmful gender norms to reduce IPV in Sri Lanka have shown promise [61]. Moreover, findings from other social norm interventions in South Africa [62] and Uganda [63] showed that a focus on community mobilisation and tackling harmful gender norms was effective in reducing IPV [63–65].

The current findings also highlight the importance of prioritising interventions in post-conflict areas. Trauma-informed approaches to addressing both IPV and suicidal behaviour should be considered, not only for populations in post-conflict settings, but for individuals experiencing IPV-related trauma. Health care professionals should be equipped to identify and respond to IPV in a safe and compassionate manner, with follow-up support provided as needed [66].

### Limitations

Area-level inferences between IPV and suicidal behaviour should be interpreted with caution due to the ecological fallacy of making individual inferences from aggregate data. Despite this, current findings show strong associations between individual-level IPV and household-level self-harm. Although the SLDHS did not collect data on individual-level self-harm, results from the current study are consistent with studies from South Asia examining domestic violence and individual-level self-poisoning [24, 27, 52]. It is also worth noting suicidal intention and lethality of the attempt were not captured by the SLDHS questionnaire and thus it was not possible to separate suicidal and non-suicidal self-harm. In addition, given the cross-sectional nature of the study, further prospective studies are needed to determine causative links. Low counts of self-harm and overall limitations in the sample size at the province level, reduced statistical power and thus crude rates should be interpreted with caution. Furthermore, given the stigma associated with suicidal behaviour and IPV, it is likely cases are under-reported. Disclosure may vary by district depending on sociocultural factors, and quality and completeness of reporting is also likely to differ across districts and police departments [67], introducing potential information bias.

## Conclusions

IPV, suicide and self-harm are significant contributors to mortality and morbidity worldwide. The strong associations between IPV and suicidal behaviour demonstrated in Sri Lanka and consistency of this evidence across LMIC and globally, signals the need to prioritise violence reduction itself and within national suicide prevention strategies. Targeted efforts and tailored approaches are also needed in post-conflict areas given the elevated rates of IPV and suicide in these communities. Prospective and qualitative studies among men and women are needed to further understand the complex interplay of poverty, exposure to violence (including psychological abuse), alcohol misuse, and other psycho-social factors with suicidal behaviour.

## Supporting information

S1 Fig Overview of data sources linked to research objectives. (DOCX)

S1 Table Intimate partner violence (IPV) prevalence, crude rates of suicide and household-level self-harm in Sri Lanka. (DOCX)

S2 Table Sensitivity analysis of suicidal behaviour (self-harm and suicide deaths combined) by a household member in Sri Lanka: Weighted distribution and adjusted associations with ever-partnered women aged 15–49 years reporting intimate partner violence in the past year. (DOCX)
