# Supplementary material for: Intimate partner violence, suicide and self-harm in Sri Lanka: Analysis of national data
Source: PLoS One. 2024 Mar 21;19(3):e0298413. doi: 10.1371/journal.pone.0298413 (PMC10956877; doi:10.1371/journal.pone.0298413)
Supplement: S1 Fig — (DOCX) [file pone.0298413.s001.docx]

**S1 Figure. Overview of data sources linked to research objectives.**

Sri Lanka DHS data (2016)

(16,

Sri Lanka Police data (2018)

(16,

18,302 women surveyed (includes question on self-harm/suicide within household)

Suicide deaths reported by district for 2018

Objective 1) Explore regional patterns in IPV, self-harm, and suicide:

IPV prevalence and self-harm rate calculated by district (DHS data)

Crude suicide rate calculated by district (Police data)

Crude rate calculated using cases of suicide as numerator and national Census data as denominator

16,390 women complete IPV module (final analytical sample for logistic regression models)

Objective 2) Associations examined between IPV and self-harm
Objective 3) Associations between IPV and self-harm/suicide (combined)
