# Supplementary material for: Intimate partner violence, suicide and self-harm in Sri Lanka: Analysis of national data
Source: PLoS One. 2024 Mar 21;19(3):e0298413. doi: 10.1371/journal.pone.0298413 (PMC10956877; doi:10.1371/journal.pone.0298413)
Supplement: S1 Table — (DOCX) [file pone.0298413.s002.docx]

**S1 Table. Intimate partner violence (IPV) prevalence, crude rates of suicide and household-level self-harm in Sri Lanka**

| Name | Area | % IPV (95% CI)^a^ | Crude suicide rate per 100,000 population (95% CI)^b^ | Crude household self-harm rate per 100,000 population (95% CI)^c^ |
| --- | --- | --- | --- | --- |
| Western | **Province** | **12.4 (11.2-13.7)** | **12.4 (11.5-13.3)** | **435.5 (258.1-688.3)** |
| Colombo | District | 14.4 (12.2-16.9) | 13.1 (11.7-14.6) |  |
| Gampaha | District | 12.8 (11.0-14.9) | 13.1 (11.6-14.6) |  |
| Kalutara | District | 8.4 (6.7-10.5) | 9.6 (8.0-11.5) |  |
| Central | **Province** | **23.7 (21.4-26.2)** | **12.9 (11.6-14.4)** | **531.9 (265.5-951.7)** |
| Kandy | District | 25.0 (21.8-28.6) | 12.1 (10.4-14.1) |  |
| Matale | District | 30.1 (25.9-34.8) | 15.2 (12.0-18.9) |  |
| Nuwara Eliya | District | 15.9 (11.8-21.0) | 13.0 (10.5-15.8) |  |
| Southern | **Province** | **16.2 (14.3-18.4)** | **15.7 (14.3-17.3)** | **400.4 (172.9-789.0)** |
| Galle | District | 21.4 (18.1-25.2) | 15.0 (12.8-17.5) |  |
| Hambantota | District | 5.8 (4.0-8.3) | 14.5 (11.7-17.8) |  |
| Matara | District | 17.9 (14.9-21.5) | 17.6 (14.9-20.7) |  |
| Northern | **Province** | **34.9 (31.6-38.3)** | **26.4 (23.4-29.6)** | **505.1 (137.6-1293.1)** |
| Jaffna | District | 39.8 (34.5-45.3) | 23.2 (19.5-27.4) |  |
| Kilinochchi | District | 50.4 (45.1-55.7) | 36.3 (26.5-48.6) |  |
| Mannar | District | 21.1 (16.3-26.8) | 14.0 (7.9-23.12) |  |
| Mullaitivu | District | 29.3 (24.5-34.6) | 47.9 (35.1-63.9) |  |
| Vavuniya | District | 18.5 (14.5-23.2) | 26.1 (19.2-34.6) |  |
| Eastern | **Province** | **35.2 (32.0-38.4)** | **18.5 (16.5-20.7)** | **672.0 (322.3-1235.9)** |
| Ampara | District | 27.1 (23.2-31.4) | 10.2 (8.0-12.9) |  |
| Batticaloa | District | 49.9 (44.6-55.1) | 29.1 (24.8-33.9) |  |
| Trincomalee | District | 29.5 (24.0-35.7) | 18.2 (14.3-22.8) |  |
| North-Western | **Province** | **12.1 (10.6-13.8)** | **20.5 (18.7-22.3)** | **826.1 (481.2-1322.6)** |
| Kurunegala | District | 10.3 (8.7-12.2) | 21.2 (19.1-23.5) |  |
| Puttalam | District | 16.5 (13.5-19.9) | 18.9 (16.1-22.2) |  |
| North-Central | **Province** | **8.3 (6.7-10.4)** | **13.3 (11.4-15.4)** | **241.6 (49.8-705.9)** |
| Anuradhapura | District | 7.7 (5.7-10.4) | 11.7 (9.6-14.1) |  |
| Polonnaruwa | District | 9.8 (7.2-13.0) | 16.7 (13.1-21.0) |  |
| Uva | **Province** | **9.2 (7.5-11.3)** | **14.1 (12.2-16.2)** | **370.4 (100.9-948.3)** |
| Badulla | District | 10.4 (8.1-13.1) | 13.3 (11.0-16.0) |  |
| Moneragala | District | 7.5 (5.2-10.8) | 15.5 (12.2-19.4) |  |
| Sabaragamuwa | **Province** | **11.9 (10.2-14.0)** | **13.8 (12.2-15.5)** | **456.6 (183.6-940.8)** |
| Kegalle | District | 9.0 (6.4-12.7) | 9.4 (7.4-11.6) |  |
| Ratnapura | District | 13.5 (11.3-16.1) | 17.1 (14.8-19.7) |  |
| National | **National** | **16.9 (16.1-17.6)** | **15.3 (14.8-15.8)** | **500.2 (397.9-620.9)** |

^a^ Data source: 2016 Sri Lanka Demographic and Health Survey.
^b^ Data source: Numerator = Sri Lanka Department of Police, Division of Statistics 2018 suicide data; Denominator = Sri Lanka Department of Census and Statistics 2017 mid-year population estimates.  ^c^ Data source: 2016 Sri Lanka Demographic and Health Survey. District-level crude self-harm rates not shown as the numbers per districts were too low to estimate meaningful rates with confidence intervals.
