# Supplementary material for: Intimate partner violence, suicide and self-harm in Sri Lanka: Analysis of national data
Source: PLoS One. 2024 Mar 21;19(3):e0298413. doi: 10.1371/journal.pone.0298413 (PMC10956877; doi:10.1371/journal.pone.0298413)
Supplement: S2 Table — (DOCX) [file pone.0298413.s003.docx]

**S2 Table. Sensitivity analysis of suicidal behaviour (self-harm and suicide deaths combined) by a household member in Sri Lanka: weighted distribution and adjusted associations with ever-partnered women aged 15-49 years reporting intimate partner violence in the past year.**

|  | Past year household self-harm and suicide | | |
| --- | --- | --- | --- |
|  | **Yes**  **N (%)** | **No**  **N (%)** | **Adjusted odds ratio* (95% CI)** |
| Any abuse |  |  |  |
| No | 44 (58.1) | 13,631 (83.3) | 1.00 |
| Yes | 32 (41.9) | 2762 (16.7) | 3.60 (2.19-5.92) |
| Physical/sexual abuse |  |  |  |
| No | 44 (63.3) | 13,631 (89.5) | 1.00 |
| Yes | 26 (36.7) | 1616 (10.5) | 4.97 (2.92-8.46) |
| Psychological abuse |  |  |  |
| No | 44 (60.3) | 13,631 (86.3) | 1.00 |
| Yes | 29 (39.7) | 2192 (13.7) | 4.18 (2.50-7.00) |
| Frequency of abuse |  |  |  |
| None | 44 (58.1) | 13,631 (83.3) | 1.00 |
| Less often | 13 (17.6) | 1476 (9.0) | 2.84 (1.46-5.53) |
| Daily/weekly/monthly | 18 (24.3) | 1286 (7.8) | 4.47 (2.46-8.13) |

*Adjusting for age. CI = Confidence interval.
